# Thirty-Day Complications, Unplanned Hospital Encounters, and Mortality after Endosonography and/or Guided Bronchoscopy: A Prospective Study

**DOI:** 10.3390/cancers15184531

**Published:** 2023-09-12

**Authors:** Daniele Magnini, Giovanni Sotgiu, Giuseppe Bello, Mariangela Puci, Vanina Livi, Antonio Maria Dell’Anna, Paolo De Santis, Ruben Dell’Ariccia, Marta Viscuso, Maria Chiara Flore, Alessandra Bisanti, Daniela Paioli, Antonio Gullì, Fausto Leoncini, Massimo Antonelli, Rocco Trisolini

**Affiliations:** 1 Interventional Pulmonology Division, Fondazione Policlinico Universitario A. Gemelli IRCCS, 00168 Rome, Italy; daniele.magnini@policlinicogemelli.it (D.M.); vanina.livi@policlinicogemelli.it (V.L.); mariachiara.flore@guest.policlinicogemelli.it (M.C.F.); daniela.paioli@policlinicogemelli.it (D.P.); fausto.leoncini@policlinicogemelli.it (F.L.); 2 Clinical Epidemiology and Medical Statistics Unit, Department of Medicine, Surgery and Pharmacy, University of Sassari, 07100 Sassari, Italy; gsotgiu@uniss.it (G.S.); pucimariangela@gmail.com (M.P.); 3 Department of Emergency, Intensive Care Medicine and Anesthesia, Fondazione Policlinico Universitario A. Gemelli IRCCS, 00168 Rome, Italy; giuseppe.bello@policlinicogemelli.it (G.B.); antoniomaria.dellanna@polilcinicogemelli.it (A.M.D.); paolo.desantis@policlinicogemelli.it (P.D.S.); alessandra.bisanti@policlinicogemelli.it (A.B.); antonio.gulli@policlinicogemelli.it (A.G.); massimo.antonelli@policlinicogemelli.it (M.A.); 4 Pulmonology Division, Fondazione Policlinico Universitario A. Gemelli IRCCS, 00168 Rome, Italy; rubendellariccia1@gmail.com (R.D.); marta.viscuso@gmail.com (M.V.); 5 Department of Cardiovascular and Pulmonary Sciences, Catholic University of the Sacred Hearth, 00168 Rome, Italy; 6 Department of Anesthesiology and Critical Care Medicine, Catholic University of the Sacred Hearth, 00168 Rome, Italy

**Keywords:** complications, endobronchial ultrasound, guided bronchoscopy, lung cancer, Charlson Comorbidity Index, low-dose attenuation, pneumonia, adverse event, mediastinitis

## Abstract

**Simple Summary:**

This study confirmed the overall safety of endosonography and guided bronchoscopy, even in a cohort of patients with multiple comorbidities and a high prevalence of advanced cancer. However, it suggested that implementing a 30-day follow-up in routine clinical practice would also help identify and treat clinically relevant late complications promptly while establishing a more realistic rate of adverse events for these procedures.

**Abstract:**

Background and objective: Limited data exist regarding the adverse events of advanced diagnostic bronchoscopy, with most of the available information derived from retrospective datasets that primarily focus on early complications. Methods: We conducted a 15-month prospective cohort study among consecutive patients undergoing endosonography and/or guided bronchoscopy under general anesthesia. We evaluated the 30-day incidence of severe complications, any complication, unplanned hospital encounters, and deaths. Additionally, we analyzed the time of onset (immediate, within 1 h of the procedure; early, 1 h–24 h; late, 24 h–30 days) and identified risk factors associated with these events. Results: Thirty-day data were available for 697 out of 701 (99.4%) enrolled patients, with 85.6% having suspected malignancy and multiple comorbidities (median Charlson Comorbidity Index (IQR): 4 (2–5)). Severe complications occurred in only 17 (2.4%) patients, but among them, 10 (58.8%) had unplanned hospital encounters and 2 (11.7%) died within 30 days. A significant proportion of procedure-related severe complications (8/17, 47.1%); unplanned hospital encounters (8/11, 72.7%); and the two deaths occurred days or weeks after the procedure. Low-dose attenuation in the biopsy site on computed tomography was independently associated with any complication (OR: 1.87; 95% CI 1.13–3.09); unplanned hospital encounters (OR: 2.17; 95% CI 1.10–4.30); and mortality (OR: 4.19; 95% CI 1.74–10.11). Conclusions: Severe complications arising from endosonography and guided bronchoscopy, although uncommon, have significant clinical consequences. A substantial proportion of adverse events occur days after the procedure, potentially going unnoticed and exerting a negative clinical impact if a proactive surveillance program is not implemented.

## 1. Introduction

Endosonography and guided bronchoscopy play a vital role in the minimally invasive diagnosis of mediastinal and pulmonary disorders. The utilization of these procedures has increased exponentially in the last decade, driven by remarkable advancements in thoracic oncology and the implementation of lung cancer screening [1,2,3]. Concurrently, the emergence of personalized oncological treatments has expanded the indication for invasive diagnostic testing to include individuals who are more susceptible to complications, such as elderly and comorbid patients with metastatic cancer [4]. Additionally, the latest advanced diagnostic techniques, especially guided bronchoscopy using modern imaging tools, are long and often complex procedures that are best performed under deep sedation or general anesthesia [5,6,7,8].

Unfortunately, while the diagnostic test characteristics of endosonography and guided bronchoscopy have understandably attracted much attention, the adverse events and downstream medical costs associated with these interventions remain largely understudied. Moreover, studies investigating complications related to these diagnostic interventions are limited by various methodological issues [9]. The low occurrence rate of adverse events necessitates large sample sizes, which can be challenging to attain in prospective settings. Although retrospective datasets can help gather larger sample sizes, they tend to underestimate complication rates due to recall bias [10,11] or under-coding when relying on administrative data [12,13,14]. Besides, as outcome measures are not defined a priori in retrospective studies, there can be wide variations as to what constitutes a complication [8]. Finally, safety is typically a secondary endpoint in most studies, and the assessment of complications is limited to a short period of time (24 h) following the procedure [11,15,16].

To address these limitations, the present study was designed to prospectively assess key composite safety outcomes, established a priori, over an extended time frame (30 days) in a large cohort of patients undergoing endosonography and/or guided bronchoscopy.

## 2. Methods

### 2.1. Study Design, Setting, and Participants

This was a single-center, prospective cohort study aimed at evaluating the adverse events occurring in the 30 days after guided bronchoscopy and/or endosonography under general anesthesia. Patients were eligible if they were ≥18 years and had an indication for guided bronchoscopy and/or endosonography for any diagnostic suspicion. Exclusion criteria included: (i) inability or unwillingness to provide consent; (ii) platelet count < 50.000 per μL; (iii) inability to stop anticoagulant or antiplatelet therapy before the procedure (except acetylsalicylic acid 100 mg/day). All the study procedures were carried out from November 2020 to January 2022 at the Interventional Pulmonology Division of the Fondazione Policlinico Universitario Agostino Gemelli IRCSS in Rome, Italy. The study was performed according to Strengthening the Reporting of Observational Studies (STROBE) guidelines.

### 2.2. Anesthesia and Monitoring Protocol

Total intravenous anesthesia was administered to all patients. Electrocardiogram, non-invasive blood pressure, and peripheral oxygen saturation were monitored throughout the bronchoscopy. A 20-gauge catheter was placed in a vein located in the hand or forearm and connected to a 3-way needle-free system, allowing the infusion of both crystalloid fluids at a constant flow of 3–4 mL/kg/h and anesthetic drugs delivered using an Alaris system infusion pump (Carefusion 303, Inc. 10020 Pacific Mesa Blvd. San Diego, CA, USA).

Preoxygenation with tidal volume breathing was performed prior to anesthetic induction for 3–5 min. General anesthesia was induced with remifentanil (0.05–0.08 μg/kg/min from the beginning of preoxygenation) and propofol (2–2.5 mg/kg). Instead of remifentanil, a bolus dose of 0.5–1 mcg/kg of fentanyl could be used, followed by repeated doses where necessary. Anesthesia was maintained with a continuous infusion of remifentanil (0.08–0.2 μg/kg/min) and propofol (3–9 mg/kg/h). Alternatively, the target controlled infusion method (TCI) was used: propofol was given using the TCI pharmacokinetic Schnider model, setting initial effect-site target concentrations at 4–6 μg/mL for induction and 2.5–4 μg/mL for maintenance of anesthesia, making repeated 0.5–1.0 μg/mL changes in the target concentration, as needed; the Minto model was used for remifentanil, with effect-site target concentrations of 1.3–1.5 ng/mL and 1.5–3 ng/mL for anesthesia induction and maintenance, respectively. Our institutional practice is to individualize doses of anesthetic agents according to age, physical status, underlying pathological condition, and type of procedure.

A supraglottic airway device (SAD; i-gel, Intersurgical Ltd., Wokingham, UK) was positioned in all patients. The correct placement of the SAD was confirmed with direct endoscopic visualization. Ventilation was carried out with a Dräger Evita XL ventilator (Drägerwerk AG & Co. KGaA Moislinger Allee 53–55 23558 Lübeck, Germany) using volume- or pressure-targeted modes.

### 2.3. Advanced Diagnostic Bronchoscopy Protocol

All endoscopic procedures were conducted by a team of 5 interventional pulmonologists, with rapid on-site evaluation (ROSE) available for each procedure.

*Endosonography:* Endobronchial ultrasound (EBUS) and endoscopic ultrasound (EUS-B) were performed using a linear ultrasound bronchoscope (BF-UC260FW, Olympus Corporation, Tokyo, Japan, or EB19-J10U, Pentax Medical, Japan). Sampling (EBUS-TBNA or EUS-B-FNA) was carried with 21-, 22-, or 25-gauge dedicated needles (Vizishot NA-201SX-4021/4022, ViziShot Flex NA-U401SX-4022, Olympus Corporation, Tokyo, Japan; ECHO-HD-22-EBUS-P, ECHO-HD-25-EBUS-P, Cook Medical Inc., Bloomington, IN, USA). For EUS-B procedures, the laryngeal mask was retrieved slightly proximally to allow for the smooth introduction of the scope into the esophagus. In patients undergoing endosonography for the mediastinal staging of lung cancer, 2 needle passes per lymph node station were deemed sufficient, as long as ROSE indicated the adequacy of the lymph node specimen. Adequacy was defined as a predominance of lymphocytes with minimal or no bronchial cells present. For patients with suspected advanced malignancy, a minimum of 4 needle passes were performed irrespective of the ROSE results. This approach aimed to obtain a sample sizable enough for subsequent immunohistochemistry studies and/or molecular profiling.

*Guided Bronchoscopy:* Guided bronchoscopy was performed with 4.2 mm and/or 3 mm outer diameter videobronchoscopes (BF-P190 and BF-MP190F, Olympus Corporation, Japan). Fluoroscopy (Ziehm Solo FD, Ziehm Imaging, Nuremberg, Germany) and radial miniature ultrasonic probes (UM-S20-17S, Olympus Corporation, Tokyo, Japan) were used as imaging methods for navigation and confirmation, respectively. Sampling was carried out with a forceps biopsy (FB-231D.A and/or FB-433D, Olympus Corporation, Japan) and/or needles (NA-1C-1 and/or NA-403D-2021, Olympus Corporation, Tokyo, Japan) under real-time fluoroscopic guidance. Bronchial washing was conducted exclusively for microbiological examinations when infection was considered in the list of possible diagnoses. In patients suspected of having locally advanced primary lung cancer, guided bronchoscopy was carried out subsequent to endosonographic mediastinal staging. For patients suspected of having advanced malignancy, guided bronchoscopy was performed first. Endosonography was then performed in cases where the ROSE of lung specimens yielded inconclusive biopsy results.

### 2.4. Data Collection

The following data were collected for each patient at the time of the procedure, concerning variables that are commonly assessed in studies examining adverse events associated with guided bronchoscopy and endosonography [15,16]: age; sex; smoking habit; body mass index (BMI); Charlson Comorbidity Index (CCI) [17,18]; American Society of Anesthesiologists (ASA) physical status; presence of fever, sputum, and dyspnea; hospital setting (inpatient vs. outpatient); indication for the procedure; presence of low-dose attenuation (LDA) in the lung and/or lymph node target lesion on computed tomography (CT) [19,20,21,22]; presence of the coagulation necrosis sign (CNS) in the target lymph node on B-mode endosonographic examination; presence of necrotic material in retrieved samples during gross examination; identification number of video bronchoscopes and/or echobronchoscopes used; type of procedure (endosonography, guided bronchoscopy, or endosonography + guided bronchoscopy); type of endosonography, if performed (EBUS, EUS-B, or EBUS + EUS-B); target sampled (lung, lymph node, or lung + lymph node); absolute number of targets sampled; procedure duration; antibiotic treatment, if any (prophylactic, during/after the procedure); final diagnosis.

### 2.5. Study Outcomes and Their Assessment

The primary endpoint was the 30-day incidence of severe complications. These were defined as clinically relevant events that either led to the premature interruption of the procedure or posed a threat to the patient’s health status. Such events encompassed active problems necessitating intervention to avert further damage (e.g., pneumonia, esophageal laceration, respiratory failure) and unforeseen occurrences that, while not causing significant harm in all instances, held substantial potential to do so (e.g., pneumothorax) [11,23,24]. Secondary endpoints included: 30-day incidence of any complication; 30-day incidence of unplanned hospital encounters (UHE—emergency department (ED) visits and/or inpatient admissions); 30-day mortality; time of onset of adverse events (immediate (within 1 h of the procedure), early (1 h to 24 h), late (1 to 30 days)); and risk factors for an adverse event.

Immediate complications were assessed during bronchoscopy and in the recovery room after the procedure. To capture early and late complications, patients were systematically contacted for a telephone interview on days 1 and 30 after the procedure. To minimize the risk of missing follow-up in the case of unforeseen events (e.g., ICU admission, death), a designated “backup” person chosen by each enrolled patient was instructed about the aims of the study and the nature of the follow-up. Furthermore, patients (and back-up persons, if needed) were encouraged to contact the investigators by telephone and/or by email at any time within 30 days after the procedure to report any change in their health status and/or any UHE. The medical charts of each outpatient diagnostic visit/work-up, as well as the ED or the hospital discharge report from patients who experienced health problems within 30 days, were systematically retrieved to obtain a reliable evaluation of the occurrence and time of onset of any adverse event possibly related to the procedure.

The assignment of an adverse event to the procedure was determined through consensus between two interventional pulmonologists, with one of them being the performing physician. In the case of a disagreement, a third interventional pulmonologist, who was unbiased to the opinions of the others, was consulted to provide a resolution.

### 2.6. Sample Size Calculation

The literature shows a percentage of severe complications in the range of 0.1–0.5% for simple bronchoscopic inspection [25], 0.15–1% for endosonography [10,11,24,26], and of 1.6–5% for guided bronchoscopy [5,6,7,15,27]. Based on such data and considering our practice pattern (65% endosonographies and 35% guided bronchoscopies), we estimated a percentage of severe complications of 1.8% in a cohort of patients submitted to endosonography and/or guided bronchoscopy. Assuming a 1.5% difference in the incidence of severe complications between simple bronchoscopic inspection and endosonography and/or guided bronchoscopy, and alpha and beta errors of 0.05 and 0.2, we calculated a sample size of 648. Considering a 3% loss to follow-up rate, a definitive sample size of 668 was estimated.

### 2.7. Statistical Analysis

Quantitative variables were summarized as median and 25th–75th percentiles (IQR), while qualitative variables were presented as absolute and relative frequencies (percentages). The Shapiro–Wilk test was used to assess the normality of the data. Pearson or Fisher exact tests were used to evaluate differences in qualitative variables, while the Mann–Whitney test was performed to compare quantitative variables. Logistic regression models were used to evaluate the relationship between demographic, epidemiological, clinical, and procedural characteristics and the following outcomes: severe complications; any complication; unplanned hospital encounters; and mortality (at 30 days). Candidate variables were selected based on their clinical or statistical significance in the univariate analysis. A *p*-value < 0.05 was considered statistically significant. Statistical computations were performed using STATA17 software.

## 3. Results

### 3.1. Patient and Procedural Characteristics

**Figure 1:**
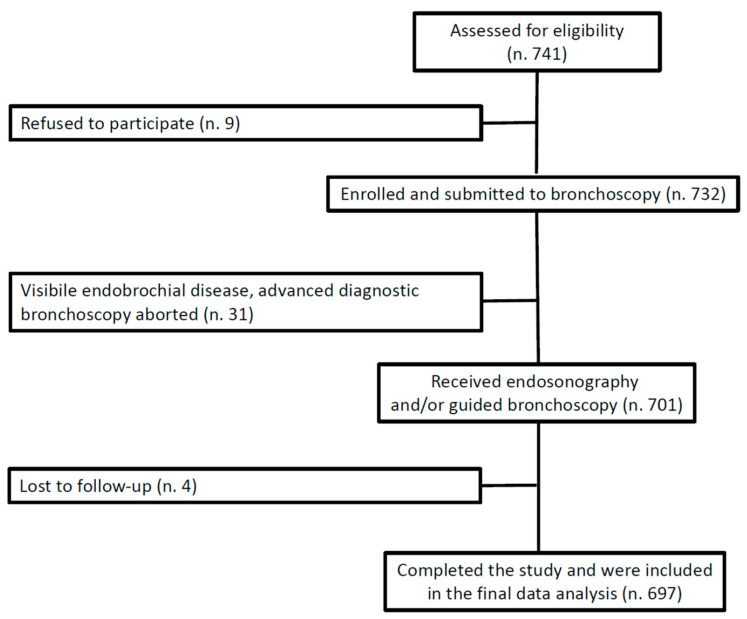
Strobe flow diagram of study enrollment.

During the study period, 701 patients were enrolled, and complete 30-day follow-up data were obtained for 697 (99.4%) (Figure 1). Table 1 presents the baseline characteristics of the study cohort and the main procedure-related aspects. Of note, the majority of patients had an underlying malignancy (85.6%) and multiple comorbidities, with a median Charlson Comorbidity Index (CCI) of 4 (IQR 2–5). Out of the 456 patients who were ultimately diagnosed with primary lung cancer, 47 (10.3%) were classified as being in stage I, 37 (8.1%) in stage II, 96 (21.1%) in stage III, and the majority in stage IV (261, 57.2%); the remaining 15 (3.3%) patients underwent endosonograhy and/or guided bronchoscopy, as they were found to have a suspected relapse of a previously treated lung cancer. The overall diagnostic yield was 92.7% for endosonography procedures and 70.2% for guided bronchoscopy.

### 3.2. Primary Outcome

Among the enrolled patients, 17 (2.4%) experienced severe complications, with respiratory failure, infections, and bleeding (grade ≥ 3 according to the Nashville Working Group (22)) being the most common (Table 2). Notably, a significant proportion of severe complications (8, 47%) occurred late, with a median of 14 days (IQR, 8–17.5) after the procedure. Of the 17 patients with severe complications, 10 (58.8%) experienced a procedure-related UHE, and 2 (11.7%) died within 30 days. The presence of low-dose attenuation (LDA) in the target lung lesion on CT (*p* < 0.0001) and antibiotic treatment started during or after the procedure (*p* < 0.0001) were more common in patients who developed severe complications compared to those who did not (Table 3).

### 3.3. Secondary Outcomes

*Any complication:* A total of 86 complications were observed in 82 (11.8%) patients, and 28 (34.1%) of these complications occurred late after the procedure (Table 2). Patients who experienced complications had a higher frequency of sputum production (*p* = 0.003), LDA in the target lung lesion (*p* = 0.04), or both in target lung and lymph node lesions on CT (*p* = 0.04), as well as the use of antibiotic treatment during or after the procedure (*p* < 0.0001) (Supplementary Materials Table S1).

*Unplanned hospital encounters (UHEs):* A total of 40 patients (5.7%) experienced a UHE at 30 days, either through an emergency department (ED) visit (4 patients) or an ED visit that led to an inpatient admission (36 patients). Most of the UHEs (29/40, 72.5%) were hospital readmissions in subjects who had undergone the endoscopic procedure as inpatients and had been discharged in the 30 days before. Procedure-related UHEs (Table 4) were relatively uncommon (11/40, 27.5%), and the majority of them (8/11, 72.7%) occurred late after the examination. Inpatient setting (*p* = 0.03), the presence of LDA in the target lung lesion at CT (*p* < 0.0001), and antibiotic treatment administered during or after the procedure (*p* < 0.0003) were significantly more frequent in patients who experienced a UHE (Table 5).

*Mortality:* Within the 30-day follow-up period, 24 patients died (3.4% all-cause mortality), but only 2 deaths were considered related to the procedure (0.29% procedure-related mortality) (Table 4). One patient developed a coma due to an air embolism during a combined endosonography and guided bronchoscopy procedure. During the ICU stay, the patient experienced respiratory failure requiring intubation and mechanical ventilation due to bilateral pneumonia. He ultimately died 28 days after the procedure, with septic shock identified as the final cause of death. The second patient died 19 days after a combined endosonography and guided bronchoscopy procedure due to an acute exacerbation of idiopathic pulmonary fibrosis (IPF). Patients who died within 30 days of the procedure had a higher CCI (*p* < 0.0001), a higher American Society of Anesthesiologists (ASA) score (*p* = 0.002), were more frequently inpatients (*p* = 0.002), and more commonly had LDA in the target lung lesion (*p* = 0.001) or in both lung and lymph node target lesions on CT (*p* = 0.04) (Table 6).

**Figure 2:**
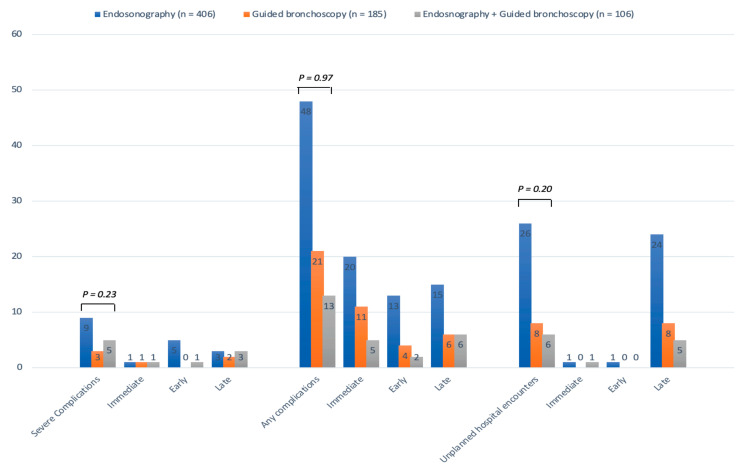
Incidence and time of onset of complications and unplanned hospital encounters according to the procedure type.

*Adverse events by procedure type:* The incidence and time of onset of severe complications, any complication, and UHEs did not significantly differ by procedure type (Figure 2). Supplementary Materials Table S2 provides a detailed breakdown of the individual complications observed for different types of procedures.

*Factors associated with adverse events:* Multivariate analysis showed a significant association (Table 7) between peri-procedural antibiotic treatment (OR: 0.25; 95% CI 0.09–0.71) and severe complications.

Sputum production (OR: 2.09; 95% CI 1.29–3.39) and LDA in the target lesion on CT (OR: 1.87; 95% CI 1.13–3.09) were associated with any complication (Supplementary Materials Table S3). UHEs were independently associated with procedure duration (OR: 0.95, 95% CI 0.93–1.00); peri-procedural antibiotic treatment (OR: 0.41, 95% CI 0.19–0.89); and LDA in the biopsy site on CT (OR: 2.17, 95% CI 1.10–4.30) (Supplementary Materials Table S4). Lastly, CCI (OR: 1.37, 95% CI 1.15–1.63); ASA score (OR: 2.89, 95% CI 1.38–6.04); and LDA in the target lesion on CT (OR: 4.19, 95% CI 1.74–10.11) were independent predictors of mortality (Table 8).

## 4. Discussion

This study assessed the overall incidence of 30-day adverse events from endosonography and guided bronchoscopy using composite safety outcomes, which have previously only been employed in the setting of therapeutic bronchoscopy [29,30].

**Figure 3:**
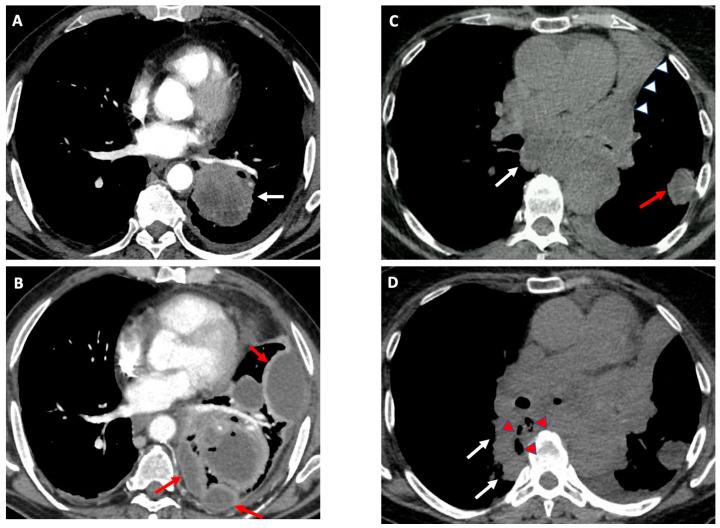
Infectious complications after endosonography: Contrast-enhanced CT scan showing a left lower lobe mass (white arrow) with low-dose attenuation (**A**). A marked increase in the size of the pulmonary mass and the presence of a multiloculated pleural effusion (red arrows) consistent with empyema were evident in a follow-up CT performed 12 days after an EBUS-TBNA of the pulmonary lesion (**B**). Unenhanced CT (patient allergic to the contrast medium) showing the complete atelectasis of the left upper lobe (with arrowheads), a subpleural nodule of the apical segment of the left lower lobe (red arrow), and an enlarged subcarinal lymph node (white arrow) (**C**). Fourteen days after an EUS-B-FNA of the subcarinal lymph node, a follow-up CT performed for the onset of persistent high-grade fever suggested a mediastinitis, as indicated by a marked increase in the size of the lymph node (white arrows) and the appearance of intralesional air coefficients (red arrowheads) (**D**).

The observed incidence of severe complications was relatively low, but their clinical impact was remarkable. Among the 17 patients who suffered from severe complications, 10 (58.8%) experienced a procedure-related UHE and 2 (11.7%) died within 30 days. Respiratory failure and infections accounted for more than half of the observed severe complications, but their clinical impact and time of onset differed (Table 2). Respiratory failure occurred within 24 h after the procedure in four out of five patients, requiring a UHE in only one case. On the other hand, infectious complications (Figure 3), which occurred only in patients with an underlying malignancy, had a lower incidence compared to previous studies [19,20,21,22] but had a significant clinical and economic impact in our cohort. Infections led to an escalation of care (unplanned hospital admission for outpatients or the substantial extension of hospital stay for inpatients), required a prolonged (>2 weeks) intravenous antibiotic treatment, and caused a significant delay in anti-cancer treatment.

The low rate of pneumothorax in our population exemplifies the limitations of relying on an individual event as a safety endpoint and has several possible explanations. Our enrollment took place mostly during the second and third waves of the COVID-19 pandemic, when resources were constrained and there was a risk of the transmission of infection to patients and staff. Therefore, we tended to prioritize advanced diagnostic bronchoscopy for patients with locally advanced or advanced malignancy and referred patients with solitary pulmonary nodules with a medium-to-high risk of malignancy directly for curative resection [31]. Additionally, we always performed lung peripheral sampling under fluoroscopy and radial EBUS guidance, which is associated with a significantly lower incidence of pneumothorax, as shown in a large study assessing the role of electromagnetic navigation bronchoscopy for the diagnosis of pulmonary lesions [5,32].

A substantial proportion of adverse events occurred days or even weeks after the procedures (Table 2), highlighting the poor awareness and underreporting of late complications of endoscopic procedures. Studies have demonstrated that physicians performing colonoscopies are unaware of nearly 75% of the hospital admissions for adverse events after the procedure [33]. Poor awareness leads to underreporting, as evidenced in patients receiving invasive testing in the context of lung cancer screening programs [12,13,14,34]. These studies, relying on administrative data at 30 or 90 days from the procedure, reported significantly higher overall and severe complication rates related to bronchoscopy compared to studies with a shorter follow-up. Underreporting is particularly evident for complications that occur late after the procedure due to their pathophysiology. For example, large studies with short follow-up periods completely missed cases of post-bronchoscopic pneumonia [15,16,35], whereas studies with a longer follow-up reported significant incidence rates (4–6.3%) (26–28). Our data strongly support the need for a proactive and longer follow-up after these procedures in everyday clinical practice. This approach would allow for the identification and earlier treatment of clinically relevant late complications, help define a realistic adverse events rate for endosonography and guided bronchoscopy, and improve patient information and endoscopy quality assessment.

UHEs have been tentatively studied as a surrogate of adverse events following outpatient procedures in the setting of GI endoscopy and have been proposed as a quality indicator with some controversy [36,37,38]. The 1.6% incidence of a procedure-related UHE at 30 days that we found after endosonography and/or guided bronchoscopy is reassuringly low and consistent with the 1.3% readmission rate recently reported after transbronchial lung cryobiopsy in the setting of interstitial lung disease [39].

The 3.4% all-cause mortality rate at 30 days was expected, considering the high prevalence of patients with multiple comorbidities and advanced malignancy in our population. However, the notable 0.29% procedure-related mortality could be explained by the long follow-up we employed and the broad definition of “procedure-related” used. The 30-day time frame allowed us to capture the two procedure-related fatalities, which occurred 20 and 29 days after the intervention and may have gone unnoticed with a shorter follow-up period (Table 4). Furthermore, we attributed to the procedure not only the death caused by an adverse event (i.e., AE-IPF), which had already been associated with bronchoscopy [40,41], but also the death ultimately caused by septic shock. The latter patient ended up in the ICU due to a severe immediate adverse event of bronchoscopy (air embolism), and the subsequent complications (bilateral pneumonia, respiratory failure, and septic shock) that developed and ultimately caused his death would likely not have occurred if he had not undergone the procedure.

Although general anesthesia is considered a potential risk factor for complications, we did not observe clinically significant anesthesia-related adverse events in our cohort, despite the high prevalence of frail patients. We recorded several cases of hypotension in the first minutes after anesthesia induction, but the vast majority of them resolved spontaneously within 5 min. This finding was consistent with prior studies in the settings of EBUS-TBNA, guided bronchoscopy, and therapeutic bronchoscopy, which showed lower complication rates when general anesthesia was used [29,32,42,43].

Among the risk factors for adverse events identified in this study, two deserve particular mention. LDA in the target lesion on CT was an independent predictor of any complication, UHEs, and death. Specifically, LDA in the target lung lesion was significantly more common in patients who developed adverse events (Figure 3). This finding was consistent with previous studies that have identified LDA as a strong predictor of infections in patients undergoing guided bronchoscopy [20,22,44]. Second, antibiotic treatment administered during or after the procedure was independently associated with severe complications and UHEs. However, we typically initiated antibiotics during/after the procedure in patients who had pre-existing risk factors for infectious complications (e.g., diabetes and low-dose attenuation in the target lesion on CT) and who were found to have additional risk factors during bronchoscopy (e.g., the presence of abundant airway purulent secretions) or developed persistent fever after the procedure. Therefore, it is plausible that these patients were inherently at a higher risk of adverse events. The use of antibiotic prophylaxis to prevent infectious complications in the presence of LDA in the target lung lesions remains a matter of debate, with conflicting results emerging from the available studies [20,21,44,45] and current guidelines not recommending their routine use [46]. Functional status and comorbidities hold remarkable prognostic significance in lung cancer [17,47]. However, a comprehensive study utilizing US AQuIRE registry data found that performance status, as assessed by the Zubrod score, did not emerge as an independent risk factor for complications associated with advanced diagnostic bronchoscopy [16]. These findings prompted us to shift our focus towards investigating the potential association of the Charlson Comorbidity Index with adverse events. Notably, the prognostic relevance of comorbidities, regardless of performance status and tumor stage, has been extensively documented across different cancer types, including lung cancer [47,48]. In our series, the Charlson comorbidity index emerged as an independent predictor of 30-day mortality, albeit not for complications.

The strengths of our study included the prospective design, relatively large sample size, and use of clearly defined safety outcomes measured over a long period. However, some limitations should be acknowledged. The study was carried out in a single, large-volume academic center by experienced operators and anesthesiologists. Additionally, the majority of patients had locally advanced or advanced malignancies. These factors may have limited the external validity of our results for populations comprising a more balanced mix of patients with early and locally advanced/advanced malignancy. Furthermore, we acknowledge that the clinical grading of some complications, as well as establishing a cause–effect relationship between the procedure and adverse events occurring days after, is subjective by nature. Finally, we simultaneously tested several variables, a practice that could potentially elevate the risk of a type I error. However, our deliberate choice of a less conservative approach was guided by the intention to strike a balance between both type I and type II errors. This approach was specifically designed to prevent the unwarranted exclusion of potentially valuable information for future studies.

## 5. Conclusions

Our study confirmed the overall safety of endosonography and guided bronchoscopy, even in a cohort of patients with multiple comorbidities and a high prevalence of advanced cancer. However, it suggested that implementing a 30-day follow-up in everyday clinical practice would facilitate the early identification and treatment of clinically relevant late complications and establish a realistic adverse events rate for these procedures.

## Figures and Tables

**Table 1:** Demographic, clinical, and procedural characteristics.

Variable	Patients (n = 697)
**Median (IQR) age, years**	68 (58–75)
** Males, n (%) **	399 (57.2)
**Smoking, n (%)**	
Current	216 (31.0)
Former	310 (44.5)
Never	171 (24.5)
** Median (IQR) BMI, kg/m^2^**	25 (22–27)
**Charlson Comorbidity Index, n (%)**	
0–1	95 (13.6)
2–3	239 (34.3)
4–5	201 (28.8)
>5	162 (23.2)
**ASA score, n (%)**	
I	139 (19.9)
II	437 (62.7)
III	115 (16.5)
IV	6 (0.9)
**Fever, n (%)**	17 (2.4)
**Dyspnea, mMRC, n (%)**	
0	91 (13.1)
I	267 (38.3)
II	233 (33.4)
III	97 (13.9)
IV	9 (1.3)
**Sputum production, n (%)**	
No	488 (70.0)
Yes, whitish	150 (21.5)
Yes, purulent	35 (5.0)
Yes, hemoptysis	24 (3.5)
**Setting, n (%)**	
Inpatient	392 (56.2)
Outpatient	305 (43.8)
**Procedure type, n (%)**	
Endosonography	406 (58.3)
Guided bronchoscopy	185 (26.5)
Endosonography + guided bronchoscopy	106 (15.2)
**Endosonography type, n (%)**	
None	185 (26.5)
EBUS	471 (67.6)
EUS-B	34 (4.9)
EBUS + EUS-B	7 (1.0)
** Median (IQR) procedure duration, minutes **	30 (23–37)
** Median (IQR) n. of biopsy targets **	1 (1–2)
**Target lesion, n (%)**	
Lymph node	327 (46.9)
Lung lesion	221 (31.7)
Lymph node + lung lesion	149 (21.4)
**LDA in target lesion at CT, n (%)**	
No	531 (76.2)
Yes, lymph node(s)	83 (11.9)
Yes, lung lesion(s)	54 (7.7)
Yes, lymph node(s) + lung lesion(s)	29 (4.2)
**CNS in target lesion at endosonography, n (%)**	46/512 (9.0)
**Peri-procedural antibiotic treatment, n (%)**	
None	609 (87.4)
Prophylactic	15 (2.1)
During/post-procedure	73 (10.5)

Abbreviations: IQR: interquartile range; BMI: body mass index; ASA: American Society of Anesthesiologists; mMRC: modified British Medical Research Council; EBUS: endobronchial ultrasound; EUS-B: endoscopic ultrasound with bronchoscope; LDA: low-dose attenuation; CT: computed tomography; CNS: coagulation necrosis sign.

**Table 2:** Detailed list of severe (17 in 17 patients) and non-severe (69 in 65 patients) complications according to their time of onset.

	Immediate		Early		Late	
**Severe complications**	Type	n	Type	n	Type	n
	Lidocaine-induced anaphylactic shock	1	Respiratory failure	4	Pulmonary infection Severe hemoptysis ^#^ Pulmonary infection and empyema Mediastinitis Acute exacerbation of pulmonary fibrosis Respiratory failure	2 2 1 1 1 1
	Air embolism	1	Pneumothorax	1		
	Bleeding grade ≥ 3 ^#^	1	Acute coronary syndrome	1		
**Mild/Moderate complications**	Type	n	Type	N	Type	n
	Laryngospasm or bronchospasm	17	Fever ^ Transient (<24 h) RF ° Persistent sore throat Vasovagal syncope Persistent headache/vomiting	7 2 2 1 1	Hemoptysis ^#^	9
	Bleeding grade ≤ 2 ^#^	16			Fever ^	7
	Transient but sustained (<80%, >5 min) hypoxemia	3			Worsening dyspnea *	4

^#^ Blood loss occurring during the procedure was referred to as “bleeding” and was graded using the Nashville Working Group scale [28]. Blood loss occurring ≥ 24 after the procedure, and not directly observed by one of the investigators, was referred to as “hemoptysis”. Hemoptysis was considered procedure-related if it occurred for the first time during endosonography and/or guided bronchoscopy and recurred within 30 days of the procedure. Hemoptysis was considered severe if it led to a UHE. ° RF: respiratory failure. * Marked worsening, after endosonography and/or guided bronchoscopy, of dyspnea already present before the procedure with no alternative cause found. ^ Fever > 38 °C occurring after the procedure, with a duration > 12 h and requiring antibiotic treatment because of a lack of response to paracetamol.

**Table 3:** Demographic, epidemiological, clinical, and procedural characteristics between 30-day severe complications groups.

Variable	No Severe Complications (n = 680)	Severe Complications (n = 17)	*p*-Value
**Median (IQR) age, years**	67 (58–75)	72 (70–75)	0.08
** Males, n (%) **	387 (56.9)	12 (70.6)	0.26
**Smoking, n (%)**			0.17
Current	210 (30.9)	6 (35.3)	
Former	300 (44.1)	10 (58.8)	
Never	170 (25.0)	1 (5.9)	
**Median (IQR) CCI, n (%)**	4 (2–5)	4 (3–5)	0.22
**Median (IQR) ASA score, n (%)**	2 (2–2)	2 (2–3)	0.30
**Fever, n (%)**	16 (2.4)	1 (5.9)	0.35
**Dyspnea, mMRC, n (%)**			0.22
0	90 (13.2)	1 (5.9)	
I	260 (38.2)	7 (41.2)	
II	229 (33.7)	4 (23.5)	
III	93 (13.7)	4 (23.5)	
IV	8 (1.2)	1 (5.9)	
**Sputum production, n (%)**			0.15
No	478 (70.3)	10 (58.8)	
Yes, whitish	145 (21.3)	5 (29.4)	
Yes, purulent	35 (5.2)	0 (0.0)	
Yes, hemoptysis	22 (3.2)	2 (11.8)	
**Setting, n (%)**			0.23
Inpatient	380 (55.9)	12 (70.6)	
Outpatient	300 (44.1)	5 (29.4)	
**Procedure type, n (%)**			0.23
Endosonography	397 (58.4)	9 (52.9)	
Guided bronchoscopy	182 (26.8)	3 (17.7)	
Endosonography + guided bronchoscopy	101 (14.9)	5 (29.4)	
**Endosonography type, n (%)**			0.66
None	182 (26.8)	3 (17.7)	
EBUS	458 (67.4)	13 (76.5)	
EUS-B	33 (4.9)	1 (5.9)	
EBUS + EUS-B	7 (1.0)	0 (0.0)	
** Median (IQR) procedure duration, minutes **	30 (23–37)	30 (27–35)	0.44
** Median (IQR) n. of biopsy targets **	1 (1–2)	2 (1–2)	0.19
**Target lesion, n (%)**			0.13
Lymph node	322 (47.4)	5 (29.4)	
Lung lesion	216 (31.8)	5 (29.4)	
Lymph node + lung lesion	142 (20.9)	7 (41.2)	
**LDA in target lesion at CT, n (%)**			
No	522 (76.8)	9 (52.9)	0.02
Yes, lymph node(s)	81 (11.9)	2 (11.8)	0.99
Yes, lung lesion(s)	49 (7.2)	5 (29.4)	<0.0001
Yes, lymph node(s) + lung lesion(s)	28 (4.1)	1 (5.9)	0.15
**CNS in target lesion at endosonography, n (%)**	44 (8.8)	2 (14.3)	0.36
**Peri-procedural antibiotic treatment, n (%)**			
None	599 (88.1)	10 (58.8)	0.0003
Prophylactic	15 (2.2)	0 (0.0)	0.54
During/post-procedure	66 (9.7)	7 (41.2)	<0.0001

Abbreviations: IQR: interquartile range; BMI: body mass index; ASA: American Society of Anesthesiologists; mMRC: modified British Medical Research Council; EBUS: endobronchial ultrasound; EUS-B: endoscopic ultrasound with bronchoscope; LDA: low-dose attenuation; CT: computed tomography; CNS: coagulation necrosis sign.

**Table 4:** Detailed description of the cause of unplanned hospital encounters and deaths recorded within 30 days after endosonography and/or guided bronchoscopy.

	Procedure-Related	n = 11	Non-Procedure-Related	n = 29
**Unplanned hospital encounters**	Respiratory failure Severe hemoptysis Pneumonia Mediastinitis Sepsis from lung abscess Pneumonia with empyema Coma due to air embolism Acute exacerbation of IPF Persistent headache/vomiting °	2 2 1 1 1 1 1 1 1	Neoplastic disease progression	10
			Cardiovascular event	6
			Pulmonary embolism	2
			Severe dysphagia	2
			Chemotherapy-induced leukopenia	1
			Urosepsis	1
			Severe gastritis	1
			Chemotherapy-induced anaphylaxis	1
			Endocrine paraneoplastic syndrome	1
			Pericardial effusion	1
			Chest pain	1
			Panic attack	1
			Subcutaneous emphysema after lung surgery	1
	**Procedure-Related**	n = 2	**Non-Procedure-Related**	n = 22
**Deaths**	Acute exacerbation of IPF	1	Underlying malignant disease progression	19
			Urosepsis	1
	Septic shock from bilateral pneumonia ^	v	End-stage hepatic disease	1
			CT-guided TTNA-induced massive hemoptysis	1

^ Pneumonia developed during the ICU stay due to an air embolism occurring within a combined endosonography + guided bronchoscopy procedure; ° Started 2 h after advanced diagnostic bronchoscopy and resolved after > 24 h.

**Table 5:** Demographic, epidemiological, clinical, and procedural characteristics between unplanned hospital encounters groups.

Variable	No Unplanned Hospital Encounters (n = 657)	Unplanned Hospital Encounters (n = 40)	*p*-Value
**Median (IQR) age, years**	67 (58–75)	71.5 (62–75)	0.07
** Males, n (%) **	375 (57.1)	24 (60.0)	0.72
**Smoking, n (%)**			
Current	203 (30.9)	13 (32.5)	
Former	290 (44.1)	20 (50.0)	0.56
Never	164 (25.0)	7 (17.5)	
**Median (IQR) CCI, n (%)**	4 (2–5)	4 (3.0–7.5)	0.05
**Median (IQR) ASA score, n (%)**	2 (2–2)	2 (2–2)	0.39
**Fever, n (%)**	16 (2.4)	1 (2.5)	0.64
**Dyspnea, mMRC, n (%)**			0.21
0	87 (13.2)	4 (10.0)	
I	255 (38.8)	12 (30.0)	
II	217 (33.0)	16 (40.0)	
III	91 (13.9)	6 (15.0)	
IV	7 (1.1)	2 (5.0)	
**Sputum production, n (%)**			0.07
No	460 (70.0)	28 (70.0)	
Yes, whitish	142 (21.6)	8 (20.0)	
Yes, purulent	35 (5.3)	0 (0.0)	
Yes, hemoptysis	20 (3.0)	4 (10.0)	
**Setting, n (%)**			0.03
Inpatient	363 (55.3)	29 (72.5)	
Outpatient	294 (44.8)	11 (27.5)	
**Procedure type, n (%)**			0.61
Endosonography	380 (57.8)	26 (65.0)	
Guided bronchoscopy	177 (26.9)	8 (20.0)	
Endosonography + guided bronchoscopy	100 (15.2)	6 (15.0)	
**Endosonography type, n (%)**			0.65
None	177 (26.9)	8 (20.0)	
EBUS	442 (67.3)	29 (72.5)	
EUS-B	31 (4.7)	3 (7.5)	
EBUS + EUS-B	7 (1.1)	0 (0.0)	
** Median (IQR) procedure duration, minutes **	30 (23–37)	26.5 (21.5–31.0)	0.02
** Median (IQR) n. of biopsy targets **	1 (1–2)	1 (1–2)	0.98
**Target lesion, n (%)**			
Lymph node	310 (47.2)	17 (42.5)	
Lung lesion	209 (31.8)	12 (30.0)	0.59
Lymph node + lung lesion	138 (21.0)	11 (27.5)	
**LDA in target lesion at CT, n (%)**			
No	509 (77.5)	22 (55.0)	0.001
Yes, lymph node(s)	76 (11.6)	7 (17.5)	0.26
Yes, lung lesion(s)	44 (6.7)	10 (25.0)	<0.0001
Yes, lymph node(s) + lung lesion(s)	28 (4.3)	1 (2.5)	0.59
**CNS in target lesion at endosonography, n (%)**	43 (9.0)	3 (9.4)	0.94
**Peri-procedural antibiotic treatment, n (%)**			
None	580 (88.3)	29 (72.5)	0.004
Prophylactic	15 (2.3)	0 (0.0)	0.33
During/post-procedure	62 (9.4)	11 (27.5)	0.0003

Abbreviations: IQR: interquartile range; BMI: body mass index; ASA: American Society of Anesthesiologists; mMRC: modified British Medical Research Council; EBUS: endobronchial ultrasound; EUS-B: endoscopic ultrasound with bronchoscope; LDA: low-dose attenuation; CT: computed tomography; CNS: coagulation necrosis sign.

**Table 6:** Demographic, epidemiological, clinical, and procedural characteristics between 30-day all-cause mortality groups.

Variable	No Mortality (n = 673)	Mortality (n = 24)	*p*-Value
**Median (IQR) age, years**	67 (58–75)	72 (68.0–77.5)	0.01
** Males, n (%) **	384 (57.1)	15 (62.5)	0.60
**Smoking, n (%)**			
Current	209 (31.1)	7 (29.2)	
Former	297 (44.1)	13 (54.2)	0.56
Never	167 (24.8)	4 (16.7)	
**Median (IQR) CCI, n (%)**	4 (2–5)	6.5 (5–8)	<0.0001
**Median (IQR) ASA score, n (%)**	2 (2–2)	2 (2–3)	0.002
**Fever, n (%)**	15 (2.2)	2 (8.3)	0.11
**Dyspnea, mMRC, n (%)**			
0	86 (12.8)	5 (20.8)	0.25
I	263 (39.1)	4 (16.7)	0.03
II	227 (33.7)	6 (25.0)	0.37
III	89 (13.2)	8 (33.3)	0.01
IV	8 (1.2)	1 (4.2)	0.20
**Sputum production, n (%)**			
No	473 (70.3)	15 (62.5)	
Yes, whitish	143 (21.3)	7 (29.2)	0.62
Yes, purulent	34 (5.1)	1 (4.2)	
Yes, hemoptysis	23 (3.4)	1 (4.2)	
**Setting, n (%)**			0.002
Inpatient	371 (55.1)	21 (87.5)	
Outpatient	302 (44.9)	3 (12.5)	
**Procedure type, n (%)**			0.96
Endosonography	392 (58.3)	14 (58.3)	
Guided bronchoscopy	179 (26.6)	6 (25.0)	
Endosonography + guided bronchoscopy	102 (15.2)	4 (16.7)	
**Endosonography type, n (%)**			0.69
None	179 (26.6)	6 (25.0)	
EBUS	455 (67.6)	16 (66.7)	
EUS-B	32 (4.8)	2 (8.3)	
EBUS + EUS-B	7 (1.0)	0 (0.0)	
** Median (IQR) procedure duration, minutes **	30 (23–37)	25.5 (20.0–31.5)	0.06
** Median (IQR) n. of biopsy targets **	1 (1–2)	1 (1–2)	0.16
**Target lesion, n (%)**			0.59
Lymph node	317 (47.1)	10 (41.7)	
Lung lesion	211 (31.4)	10 (41.7)	
Lymph node + lung lesion	145 (21.5)	4 (16.7)	
**LDA in target lesion at CT, n (%)**			
No	520 (77.3)	11 (45.8)	0.0004
Yes, lymph node(s)	79 (11.7)	4 (16.7)	0.46
Yes, lung lesion(s)	48 (7.1)	6 (25.0)	0.001
Yes, lymph node(s) + lung lesion(s)	26 (3.9)	3 (12.5)	0.04
**CNS in target lesion at endosonography, n (%)**	44 (8.9)	2 (11.1)	0.75
**Peri-procedural antibiotic treatment, n (%)**			0.28
None	590 (87.7)	19 (79.2)	
Prophylactic	14 (2.1)	1 (4.2)	
During/post-procedure	69 (10.3)	4 (16.7)	

Abbreviations: IQR: interquartile range; BMI: body mass index; ASA: American Society of Anesthesiologists; mMRC: modified British Medical Research Council; EBUS: endobronchial ultrasound; EUS-B: endoscopic ultrasound with bronchoscope; LDA: low-dose attenuation; CT: computed tomography; CNS: coagulation necrosis sign.

**Table 7:** Logistic regression analysis to assess the relationship between demographic, clinical, and procedural characteristics and 30-day severe complications.

Variables	Univariate Analysis		Multivariate Analysis	
	OR 95% CI	*p*-Value	OR 95% CI	*p*-Value
**Age, years**	1.04 (0.99–1.09)	0.09	1.03 (0.99–1.09)	0.18
**Males**	1.82 (0.63–5.21)	0.27	1.56 (0.53–4.64)	0.42
**Smoking habit**				
Never	Ref.	Ref.	Ref.	Ref.
Current	4.86 (0.58–40.74)	0.15	-	-
Former	5.67 (0.72–44.65)	0.10	-	-
**CCI**	1.12 (0.93–1.34)	0.24	-	-
**ASA score**	1.62 (0.77–3.39)	0.20	-	-
**Fever**				
Yes	Ref.	Ref.	Ref.	Ref.
No	0.39 (0.05–3.09)	0.37	-	-
**Sputum production**	1.66 (0.62–4.41)	0.31	-	-
**Setting**				
Outpatient	Ref.	Ref.	Ref.	Ref.
Inpatient	1.89 (0.66–5.44)	0.24	-	-
**Procedure type**				
Endosonography + guided bronchoscopy	Ref.	Ref.	Ref.	Ref.
Endosonography	0.46 (0.15–1.40)	0.17	-	-
Guided bronchoscopy	0.33 (0.08–1.42)	0.14	-	-
**Endosonography type**				
None	Ref.	Ref.	Ref.	Ref.
EBUS/EUS-B/EBUS-EUS-B	1.71 (0.49–6.00)	0.41	-	-
**Procedure duration, min**	1.02 (0.98–1.06)	0.35	-	-
**N. of biopsy targets**	0.17 (0.73–1.87)	0.51	-	-
**Target lesion**				
Lymph node	Ref.	Ref.	Ref.	Ref.
Lung lesion	1.49 (0.43–5.21)	0.53	-	-
Lymph node + lung lesion	3.17 (0.99–10.17)	0.05	-	-
**N. of sampled lymph nodes**	0.99 (0.62–1.57)	0.95	-	-
**N. of sampled lung lesions**	1.82 (0.72–4.59)	0.21	-	-
**N. of needle passes**	1.00 (0.83–1.21)	0.99	-	-
**LDA in target lesion at CT**				
Lymph node/lung/both	2.94 (1.12–7.74)	0.003	1.95 (0.69–5.49)	0.21
**CNS in target lesion at endosonography**				
Yes	Ref.	Ref.	Ref.	Ref.
No	0.58 (0.13–2.68)	0.49	-	-
**Peri-procedural antibiotic treatment**				
Yes	Ref.	Ref.	Ref.	Ref.
No	0.19 (0.07–0.52)	0.001	0.25 (0.09–0.71)	0.01

Abbreviations: CCI: Charlson Comorbidity Index; ASA: American Society of Anesthesiologists; EBUS: endobronchial ultrasound; EUS-B: endoscopic ultrasound with bronchoscope; LDA: low-dose attenuation; CT: computed tomography; CNS: coagulation necrosis sign.

**Table 8:** Logistic regression analysis to assess relationship between demographic, clinical, and procedural characteristics and 30-day mortality.

Variables	Univariate Analysis		Multivariate Analysis	
	OR 95% CI	*p*-Value	OR 95% CI	*p*-Value
**Age, years**	1.05 (1.01–1.10)	0.02	0.99 (0.95–1.04)	0.78
**Males**	1.25 (0.54–2.91)	0.60	0.71 (0.28–1.82)	0.47
**Smoking habit**				
Never	Ref.	Ref.	Ref.	Ref.
Current	1.40 (1.40–4.86)	0.60	-	-
Former	1.83 (0.59–5.69)	0.30	-	-
**CCI**	1.42 (1.23–1.65)	<0.0001	1.37 (1.15–1.63)	<0.0001
**ASA score**	2.91 (1.57–5.39)	0.001	2.89 (1.38–6.04)	0.005
**Fever**				
Yes	Ref.	Ref.	Ref.	Ref.
No	0.25 (0.05–1.16)	0.08	-	-
**Dyspnea (mMRC)**				
0	Ref.	Ref.	Ref.	Ref.
1	0.26 (0.07–1.00)	0.0	-	-
2	0.46 (0.14–1.53)	0.2	-	-
3–4	2.0 (0.52–4.95)	0.42	-	-
**Sputum production**	1.42 (0.61–3.30)	0.42	-	-
**Setting, No. (%)**				
Outpatient	Ref.	Ref.	Ref.	Ref.
Inpatient	5.70 (1.68–19.29)	0.005	2.62 (0.71–9.65)	0.15
**Procedure type**				
Endosonography + guided bronchoscopy	Ref.	Ref.	Ref.	Ref.
Endosonography	0.91 (0.29–2.83)	0.87	-	-
Guided bronchoscopy	0.86 (0.24–3.10)	0.81	-	-
**Endosonography type**				
None	Ref.	Ref.	Ref.	Ref.
EBUS/EUS-B/EBUS-EUS-B	1.09 (0.42–2.78)	0.86	-	-
**Procedure duration, min**	0.96 (0.92–1.01)	0.09	-	-
**N. of biopsy targets**	1.83 (0.50–1.37)	0.46	-	-
**Target lesion**				
Lymph node	Ref.	Ref.	Ref.	Ref.
Lung lesion	1.50 (0.62–3.67)	0.37	-	-
Lymph node + lung lesion	0.85 (0.27–2.84)	0.82	-	-
**N. of sampled lymph nodes**	0.80 (0.52–1.23)	0.30	-	-
**N. of sampled lung lesions**	1.35 (0.62–2.92)	0.45	-	-
**N. of needle passes**	0.95 (0.80–1.12)	0.51	-	-
**LDA in target lesion at CT**				
Lymph node/lung/both	4.02 (1.76–9.15)	0.001	4.19 (1.74–10.11)	0.001
**CNS in target lesion at endosonography**				
Yes	Ref.	Ref.	Ref.	Ref.
No	0.78 (0.17–3.51)	0.75	-	-
**Peri-procedural antibiotic treatment**				
Yes	Ref.	Ref.	Ref.	Ref.
No	0.54 (0.19–1.47)	0.23	-	-

Abbreviations: CCI: Charlson Comorbidity Index; ASA: American Society of Anesthesiologists; mMRC: modified British Medical Research Council; EBUS: endobronchial ultrasound; EUS-B: endoscopic ultrasound with bronchoscope; LDA: low-dose attenuation; CT: computed tomography; CNS: coagulation necrosis sign.

## Data Availability

The data presented in this study are available upon reasonable request from the corresponding author.

## Supplementary Materials

The following supporting information can be downloaded at: https://www.mdpi.com/article/10.3390/cancers15184531/s1, Table S1: Demographic, epidemiological, clinical and procedural characteristics between 30-day complications groups; Table S2: Detailed list of severe and non-severe complications by procedure type; Table S3: Logistic regression analysis to assess relationship between demographic, clinical, procedural characteristics, and 30-day complications.
