# Supplementary material for: Thirty-Day Complications, Unplanned Hospital Encounters, and Mortality after Endosonography and/or Guided Bronchoscopy: A Prospective Study
Source: Cancers (Basel). 2023 Sep 12;15(18):4531. doi: 10.3390/cancers15184531 (PMC10526926; doi:10.3390/cancers15184531)
Supplement: Supplementary file 1 [file cancers-15-04531-s001.zip › cancers-2535568-supplementary.pdf]

Table S1. Demographic, epidemiological, clinical and procedural characteristics between 30-day complications groups.

Variable	No complications (n = 615)	Complications (n = 82)	p-value
**Median (IQR) age, years**	67 (58-75)	68.5 (56-75)	0.81
**Males, No. (%)**	350 (56.9)	49 (59.8)	0.63
**Smoking, No. (%)**			
Current	189 (30.7)	27 (32.9)	0.99
Former	275 (44.7)	35 (42.7)	
Never	151 (24.6)	20 (24.4)	
**Median (IQR) CCI, No. (%)**	4 (2-5)	4 (2-5)	0.88
**Median (IQR) ASA score, No. (%)**	2 (2-2)	2 (2-2)	0.64
**Fever, No. (%)**	13 (2.1)	4 (4.9)	0.13
**Dyspnea, mMRC, No. (%)**			
0	83 (13.5)	8 (9.8)	
I	241 (39.2)	26 (31.7)	0.32
II	202 (32.9)	31 (37.8)	
III	81 (13.2)	16 (19.5)	
IV	8 (1.3)	1 (1.2)	
**Sputum production, No. (%)**			
No	442 (71.9)	46 (56.1)	0.003
Yes, whitish	122 (19.8)	28 (34.2)	0.003
Yes, purulent	32 (5.2)	3 (3.7)	0.55
Yes, hemoptysis	19 (3.1)	5 (6.1)	0.16
**Setting, No. (%)**			
Inpatient	344 (55.9)	48 (58.5)	0.66
Outpatient	271 (44.1)	34 (41.5)	
**Procedure type, No. (%)**			
Endosonography	358 (58.2)	48 (58.5)	0.97
Guided bronchoscopy	164 (26.7)	21 (25.6)	
Endosonography + Guided bronchoscopy	93 (15.1)	13 (15.9)	
**Endosonography type, No. (%)**			
None	164 (26.7)	21 (25.6)	
EBUS	415 (67.5)	56 (68.3)	0.96
EUS-B	30 (4.9)	4 (4.9)	
EBUS + EUS-B	6 (1.0)	1 (1.2)	
**Median (IQR) procedure duration, minutes**	29 (23-37)	31.5 (27-35)	0.13
**Median (IQR) n. of biopsy targets**	1 (1-2)	1 (1-2)	0.44
**Target lesion, No. (%)**			
Lymph node	293 (47.6)	34 (41.5)	
Lung lesion	194 (31.5)	27 (32.9)	0.49
Lymph node + lung lesion	128 (20.8)	21 (25.6)	
**LDA in target lesion at CT, No. (%)**			
No	477 (77.6)	54 (65.9)	0.02
Yes, lymph node(s)	73 (11.9)	10 (12.2)	0.93
Yes, lung lesion(s)	43 (7.0)	11 (13.4)	0.04
Yes, lymph node(s) + lung lesion(s)	22 (3.6)	7 (8.5)	0.04
**CNS in target lesion at endosonography, No. (%)**	37 (8.2)	9 (14.8)	0.09
**Peri-procedural antibiotic treatment, No. (%)**			
None	542 (88.1)	67 (81.7)	0.10
Prophylactic	15 (2.4)	0 (0.0)	0.15
During/Post-procedure	58 (9.4)	15 (18.3)	0.01

Abbreviations: IQR: interquartile range; BMI: Body Mass Index; ASA: American Society of Anesthesiologists; mMRC: modified British Medical Research Council; EBUS: Endobronchial Ultrasound; EUS-B: Endoscopic Ultrasound with Bronchoscope; LDA: low-dose attenuation; CT: computed tomography; CNS: coagulation necrosis sign.

Table S2. Detailed list of severe and non-severe complications by procedure type

Variable	Endosonography		Guided bronchoscopy		Endosonography + guided bronchoscopy	
**Severe complications**	Type	n	Type	n	Type	n
	Respiratory failure	5	Bleeding grade ≥ 3[#]	1	Air embolism	1
	Acute coronary syndrome	1	Pulmonary infection	1	Pneumothorax	1
	Pulmonary infection	1	Severe hemoptysis[§]	1	Pulmonary infection with empyema	1
	Mediastinitis	1			Severe hemoptysis[§]	1
	Lidocaine-induced anaphylactic shock	1			Acute exacerbation of pulmonary fibrosis	1
**Mild/Moderate complications**	Type	n	Type	n	Type	n
	Laryngospasm or bronchospasm	12	Bleeding grade ≤ 2	8	Laryngospasm or bronchospasm	3
	Bleeding grade ≤ 2	6	Laryngospasm or bronchospasm	2	Bleeding grade ≤ 2	2
	Fever > 8 h	5	Hemoptysis	2	Hemopthysis	2
	Hemoptysis	5	Worsening dyspnea^	2	Fever > 8 h	1
	Fever	5	Transient but sustained (<80%, >5 min) hypoxia	1	Fever	1
	Worsening dyspnea^	2	Fever > 8 h	1		
	Transient but sustained (<80%, >5 min) hypoxia	2	Transient (< 24 h) respiratory failure	1		
	Transient (< 24 h) respiratory failure	1	Persistent sore throat	1		
	Persistent sore throat	1	Vasovagal syncope	1		
	Persistent headache/vomiting	1	Fever	1		

[#] Blood loss occurring during the procedure was referred to as "bleeding" and was graded using the Nashville Working Group scale [28]. Blood loss occurring ≥24 after the procedure, and not directly seen by one of the investigators, was referred to as "hemoptysis". Hemoptysis was considered procedure-related if it occurred for the first time during endosonography and/or guided bronchoscopy and recurred within 30 days of the procedure. Hemoptysis was considered severe if it led to an UHE.

* Marked worsening, after endosonography and/or guided bronchoscopy, of dyspnea already present before the procedure with no alternative cause found.

^ Fever > 38°C occurring after the procedure, with duration > 12 hours and requiring antibiotic treatment because of lack of response to paracetamol.

Table S3. Logistic regression analysis to assess relationship between demographic, clinical, procedural characteristics, and 30-day complications.

Variables	Univariate analysis		Multivariate analysis	
	OR 95% CI	p-value	OR 95% CI	p-value
**Age, years**	1.00 (0.89-1.02)	0.85	0.99 (0.97-1.01)	0.49
**Males**	1.12 (0.70-1.80)	0.63	1.02 (0.63-1.65)	0.95
**Smoking habit**				
Never	Ref.	Ref.	Ref.	Ref.
Current	1.09 (0.58-2.00)	0.15	-	-
Former	0.96 (0.54-1.72)	0.89	-	-
**CCI**	1.00 (0.91-1.09)	0.94	-	-
**ASA score**	1.13 (0.78-1.61)	0.53	-	-
**Fever**				
Yes	Ref.	Ref.	Ref.	Ref.
No	0.42 (0.13-1.32)	0.14	-	-
**Dyspnea (mMRC)**				
0	Ref.	Ref.	Ref.	Ref.
1	1.12 (0.48-2.57)	0.79	-	-
2	1.59 (0.70-3.61)	0.27	-	-
3-4	1.98 (0.81-4.84)	0.13	-	-
**Sputum production**	2.0 (1.25-3.20)	0.004	2.09 (1.29-3.39)	0.003
**Setting**				
Outpatient	Ref.	Ref.	Ref.	Ref.
Inpatient	1.11 (0.70-1.78)	0.66	-	-
**Underlying disease**				
Other	Ref.	Ref.	Ref.	Ref.
Malignancy	0.88 (0.46-1.65)	0.68	-	-
**Procedure type**				
Endosonography + Guided	Ref.	Ref.	Ref.	Ref.
bronchoscopy Endosonography	0.96 (0.49-1.85)	0.90	-	-
Guided bronchoscopy	0.92 (0.44-1.91)	0.82	-	-
**Endosonography type**				
None	Ref.	Ref.	Ref.	Ref.
EBUS/EUS-B/EBUS-EUS-B	1.06 (0.62-1.79)	0.84	-	-
**Procedure duration, min**	1.01 (0.99-1.03)	0.33	-	-
**N. of biopsy targets**	1.10 (0.87-1.40)	0.42	-	-
**Target lesion**				
Lymph node	Ref.	Ref.	Ref.	Ref.
Lung lesion	1.20 (0.70-2.05)	0.51	-	-
Lymph node + lung lesion	1.41 (0.70-2.05)	0.24	-	-
**N. of sampled lymph nodes**	1.01 (0.84-2.02)	0.91	-	-
**N. of sampled lung lesions**	1.30 (0.72-4.59)	0.24	-	-
**N. of needle passes**	1.04 (0.95-1.14)	0.34	-	-
**LDA in target lesion at CT**				
lymph node/lung/both	1.79 (1.09-2.94)	0.02	1.87 (1.13-3.09)	0.01
**CNS in target lesion at endosonography**				
Yes	Ref.	Ref.	Ref.	Ref.
No	0.52 (0.24-1.13)	0.10	-	-
**Peri-procedural antibiotic treatment**				
Yes	Ref.	Ref.	Ref.	Ref.
No	0.60 (0.33-1.11)	0.10	-	-

Abbreviations: CCI: Charlson Comorbidity Index; ASA: American Society of Anesthesiologists; mMRC: modified British Medical Research Council; EBUS: Endobronchial Ultrasound; EUS-B: Endoscopic Ultrasound with Bronchoscope; LDA: low-dose attenuation; CT: computed tomography; CNS: coagulation necrosis sign.

Table S3. Logistic regression analysis to assess relationship between demographic, clinical, procedural characteristics, and unplanned hospital encounters.

Variables	Univariate analysis		Multivariate analysis	
	OR 95% CI	p-value	OR 95% CI	p-value
**Age, years**	1.03 (1.00-1.06)	0.06	1.01 (0.97-1.04)	0.67
**Males**	1.13 (0.59-2.16)	0.72	0.96 (0.48-1.89)	0.89
**Smoking habit**				
Never	Ref.	Ref.	Ref.	Ref.
Current	1.50 (0.59-3.85)	0.40	-	-
Former	1.62 (0.67-3.90)	0.26	-	-
**CCI**	1.16 (1.03-1.31)	0.01	1.09 (0.95-1.26)	0.24
**ASA score**	1.24 (0.77-2.10)	0.34	-	-
**Fever**				
Yes	Ref.	Ref.	Ref.	Ref.
No	0.97 (0.13-7.53)	0.98	-	-
**Dyspnea (mMRC)**				
0	Ref.	Ref.	Ref.	Ref.
1	1.02 (0.32-3.26)	0.97	-	-
2	1.60 (0.52-4.93)	0.41	-	-
3-4	1.78 (0.52-6.10)	0.36	-	-
**Sputum production**	1.00 (1.50-2.01)	1.00	-	-
**Setting**				
Outpatient	Ref.	Ref.	Ref.	Ref.
Inpatient	2.14 (1.05-4.34)	0.04	1.61 (0.74-3.50)	0.23
**Procedure type**				
Endosonography + Guided	Ref.	Ref.	Ref.	Ref.
bronchoscopy Endosonography	1.40 (0.46-2.85)	0.78	-	-
Guided bronchoscopy	0.75 (0.25-2.23)	0.61	-	-
**Endosonography type**				
None	Ref.	Ref.	Ref.	Ref.
EBUS/EUS-B/EBUS-EUS-B	1.48 (0.67-3.26)	0.34	-	-
**Procedure duration, min**	0.96 (0.93-1.00)	0.03	0.95 (0.93-1.00)	0.04
**N. of biopsy targets**	0.93 (0.65-1.34)	0.71	-	-
**Target lesion**				
Lymph node	Ref.	Ref.	Ref.	Ref.
Lung lesion	1.05 (0.49-2.24)	0.91	-	-
Lymph node + lung lesion	1.45 (0.66-3.19)	0.35	-	-
**N. of sampled lymph nodes**	0.90 (0.65-1.23)	0.50	-	-
**N. of sampled lung lesions**	1.23 (0.67-2.26)	0.50	-	-
**N. of needle passes**	1.01 (0.89-1.15)	0.85	-	-

**LDA in target lesion at CT**				
lymph node/lung/both	2.81 (1.47-5.39)	0.002	2.17 (1.10-4.30)	0.03
**CNS in target lesion at endosonography**				
Yes	Ref.	Ref.	Ref.	Ref.
No	0.95 (0.28-3.25)	0.94	-	-
**Peri-procedural antibiotic treatment**				
Yes	Ref.	Ref.	Ref.	Ref.
No	0.35 (0.17-0.73)	0.005	0.41 (0.19-0.89)	0.03

Abbreviations: CCI: Charlson Comorbidity Index; ASA: American Society of Anesthesiologists; mMRC: modified British Medical Research Council; EBUS: Endobronchial Ultrasound; EUS-B: Endoscopic Ultrasound with Bronchoscope; LDA: low-dose attenuation; CT: computed tomography; CNS: coagulation necrosis sign.
